# Experimental violation of local causality in a quantum network

**DOI:** 10.1038/ncomms14775

**Published:** 2017-03-16

**Authors:** Gonzalo Carvacho, Francesco Andreoli, Luca Santodonato, Marco Bentivegna, Rafael Chaves, Fabio Sciarrino

**Affiliations:** 1 Dipartimento di Fisica - Sapienza Università di Roma, P.le Aldo Moro 5, I-00185 Roma, Italy; 2 International Institute of Physics, Federal University of Rio Grande do Norte, 59070-405 Natal, Brazil; 3 Institute for Theoretical Physics, University of Cologne, 50937 Cologne, Germany

## Abstract

Bell's theorem plays a crucial role in quantum information processing and thus several experimental investigations of Bell inequalities violations have been carried out over the years. Despite their fundamental relevance, however, previous experiments did not consider an ingredient of relevance for quantum networks: the fact that correlations between distant parties are mediated by several, typically independent sources. Here, using a photonic setup, we investigate a quantum network consisting of three spatially separated nodes whose correlations are mediated by two distinct sources. This scenario allows for the emergence of the so-called non-bilocal correlations, incompatible with any local model involving two independent hidden variables. We experimentally witness the emergence of this kind of quantum correlations by violating a Bell-like inequality under the fair-sampling assumption. Our results provide a proof-of-principle experiment of generalizations of Bell's theorem for networks, which could represent a potential resource for quantum communication protocols.

## Abstract (web summary)

Bell's theorem has important implications for quantum information processing. Here the authors experimentally investigate the violation of a Bell-like inequality in the case of distant parties whose correlations are mediated by independent sources, a realistic feature in future quantum networks.

As demonstrated by the celebrated Bell's theorem^1^, correlations arising from experiments with distant quantum mechanical systems are at odds with one of our most intuitive scientific notions, that of local realism. The assumption of realism formalizes the idea that physical quantities have well-defined values independently of whether they are measured or not. In turn, local causality posits that correlations between distant particles can only originate from causal influences in their common past. These two rather natural assumptions together imply strict constraints on the empirical correlations that are compatible with them. These are the famous Bell inequalities, which have been recently violated in a series of loophole-free experiments^2^,^3^,^4^ and thus conclusively established the phenomenon known as Bell non-locality. Apart from their profound implications in our understanding of nature, such experiments provide a proof-of-principle for practical applications of non-local correlations, most notably in the context of quantum networks^5^,^6^,^7^.

In a quantum network, short-distance nodes are connected by sources of entangled systems which can, via an entanglement swapping protocol^8^,^9^, establish entanglement across long distances as well. Importantly, such long-distance entanglement can in principle also be used to violate a Bell inequality and thus establish a secure communication channel^10^,^11^,^12^. Clearly, for these and many other potential applications^13^,^14^,^15^,^16^, the certification of non-local correlations across the network will be crucial. The problem, however, resides on the fact that experimental imperfections accumulate very rapidly as the size of the network and the number of sources of states increase, making the detection of Bell non-locality very difficult or even impossible by usual means^17^,^18^. One of the difficulties stems from the derivation of Bell inequalities themselves, where it is implicitly assumed that all the correlations originate at a single common source (see Fig. 1b), the so-called local hidden variable (LHV) models. Notwithstanding, in a network a precise description must take into account that there are several and independent sources of states (see Fig. 1c), which introduce additional structure to the set of classically allowed correlations. In fact, there are quantum correlations that can emerge in networks that, while admitting a LHV description, are incompatible with any classical description where the independence of the sources is considered^19^,^20^,^21^,^22^,^23^,^24^,^25^. For instance, a network with two independent sources allow for the emergence of a different kind of non-local correlations violating the so-called bilocal causality assumption^19^,^20^.

The aim of this study is to experimentally observe this different type of Bell non-locality. We experimentally implemented, using pairs of polarization-entangled photons, the simplest possible quantum network akin to a three-partite entanglement swapping scheme (see Fig. 1c). Two distant parties, Alice and Charlie, perform analysis measurements over two photons (1 and 4, see Fig. 2), which were independently generated in two different sources, whereas a third station, Bob, performs a Bell-state measurement over the two other photons (2 and 3), one entangled with Alice's photon and the other entangled with Charlie's one. This scheme allows us to observe Bell non-bilocal correlations by violating the Bell-like inequality proposed in refs 19, 20. Further, showing that our experimental data is nevertheless compatible with usual LHV models where the independence of the sources is not taken into account, we can conclude that the quantum correlations we generate across the network are truly of a different kind. Moreover, we experimentally show that beyond a certain noise threshold one can enter a region where no standard local causality violation can be extracted from the shared state between Alice and Charlie after entanglement swapping and, nevertheless, the correlations of the entire network can still violate the bilocal causality assumption.

## Results

### Local and bilocal correlations in a tripartite scenario

**Figure 1:**
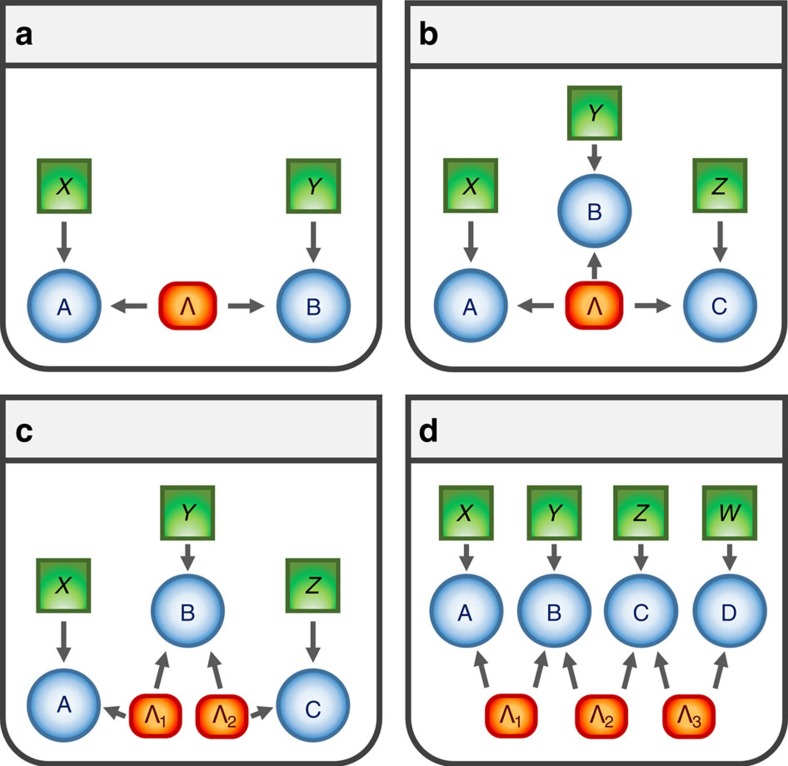
Representation of the causal structures underlying the networks^45^. Directed acyclic graphs^45^ can represent different causal structures, for instance the nodes in the graph represent the relevant random variables with arrows accounting for their causal relations. There are three different kinds of nodes: hidden variables (orange boxes), measurement settings (green boxes) and measurement outcomes (blue boxes). (**a**) Bipartite LHV model. (**b**) Tripartite LHV model. (**c**) Tripartite scenario with two independent LHVs, that is, bilocal hidden variable (BLHV) model. (**d**) Possible extension of the bilocal model to a linear chain of four stations with three independent LHVs.

We start describing the typical scenario of interest in the study of Bell non-locality shown in Fig. 1b for the case of three distant parties. A source distributes a physical system to each of the parties that at each run of the experiment can perform the measurement of different observables (labelled by *x*, *y* and *z*), thus obtaining the corresponding measurement outcomes (labelled by *a*, *b* and *c*). In a classical description of such experiment, no restrictions other than local realism are imposed, meaning that the measurement devices are treated as black boxes that take random (and independently generated) classical bits as inputs and produce classical bits as outputs as well. After a sufficient number of experimental runs is performed, the probability distribution of their measurements can be estimated, that according to the assumption of local realism can be decomposed as a LHV model of the form


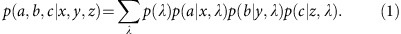


The hidden variable *λ* subsumes all the relevant information in the physical process and thus includes the full description of the source producing the particles as well as any other relevant information for the measurement outcomes.

In the description of the LHV model (equation (1)), no mention is made about how the physical systems have been produced at the source. For the network we consider here (see Fig. 1c), the two sources produce states independently, thus the set of classically allowed correlations


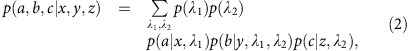


is now mediated via two independent hidden variables *λ*_1_ and *λ*_2_ (ref. 19), thus defining a bilocal hidden variable model.

In our scheme, Bob always performs the same measurement (no measurement choice) obtaining four possible outcomes that can be parameterized by two bits *b*_0_ and *b*_1_. Alice and Charlie can choose each time one of two possible dichotomic measurements. Thus, in this case the observable distribution containing the full information of the experiment is given by *p*(*a*, *b*_0_, *b*_1_, *c*|*x*, *z*). This allows us to violate the bilocal causality inequality proposed in ref. 19 and further developed in refs 20, 22, 23, 24, 25:


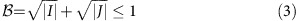


The terms *I* and *J* are sums of expectation values, given by 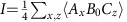 and 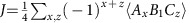, where 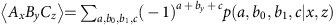 and *x*, *z*, *a*, *b*_0_, *b*_1_, *c*=0, 1. Inequality (equation (3)) is valid for any classical model of the form (equation (2)) and its violation demonstrates the non-local character of the correlations we produce among the network.

### Violation of the bilocal causality inequality

**Figure 2:**
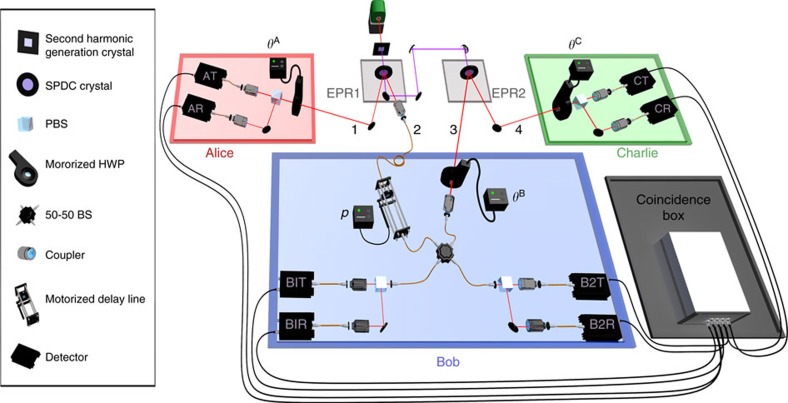
Experimental apparatus for the violation of bilocal causality. Two polarization-entangled photon pairs are generated via Spontaneous Parametric Down-Conversion (SPDC) in two separated nonlinear crystals. Photon 1 (4) of the first (second) pair is directed to Alice's (Charlie's) station, where one of the local observables *A*_0_, *A*_1_ (*C*_0_, *C*_1_) is measured via a motorized half-wave plate (HWP) (angles *θ*^A^ and *θ*^C^) followed by a polarizing BS (PBS). Photons 2 and 3 are sent to Bob's station, where a complete Bell-state measurement is performed. A 50/50 in-fibre BS followed by two PBSs allows to discriminate 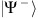 and 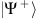 when the HWP angle *θ*^B^ is set to 0 and discriminate 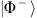 and 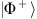 when *θ*^B^=45°. A motorized delay line is adopted to control the amount of noise *p* in the Bell measurement, by changing the photons wavepacket temporal overlap in the BS.

We generate entangled photon pairs via type-II spontaneous parametric down-conversion process occurring in two separated nonlinear crystals (Einstein-Podolsky-Rosen (EPR) 1 and EPR 2) injected by a pulsed pump laser (see Fig. 2). When a pair of photons is generated in each of the crystals, one photon from source EPR 1 (EPR 2) is sent to Alice's (Charlie's) measurement station, where polarization analysis in a basis that can be rotated of an arbitrary angle *θ*^A^ (*θ*^C^) is performed (see Supplementary Note 1). The other two photons (2 and 3) are sent to Bob's station, which consists of an in-fibre 50/50 beam splitter (BS) followed by two polarizing BSs for the polarization analysis of each of the outputs. In the ideal case (which relies on perfect photons' indistinguishability), an incoming 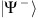 (singlet) state will feature antibunching, giving rise to coincidence counts at different outputs of the BS. All the other cases (triplet states) will experience bosonic bunching, ending up in the same BS output (see Supplementary Note 1). A twofold coincidence corresponding to different polarizations in a single BS output branch corresponds to 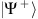 detection. A half-wave plate placed before one of the arms of the BS allows, by setting *θ*^B^=45°, to change the incoming state from 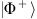 to 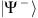 and from 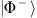 to 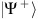. In this way, depending on the setting *θ*^B^, we are able to detect either 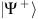 and 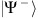 or 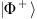 and 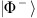 states. This detection can be interpreted as a probabilistic Bell-state measurement, where, for each pair of incoming photons, only two out of four outcomes can be unambiguously identified.

In the ideal case, Bob should be able to distinguish between all of the four Bell states, but this cannot be done by means of linear optics^26^. By this approach, however, we are able to measure all the combinations (*A*_0_, *C*_0_),(*A*_0_, *C*_1_),(*A*_1_, *C*_0_),(*A*_1_, *C*_1_) of the observables 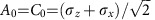 and 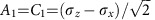 of Alice and Charlie, for the two possible *θ*^B^ configurations. The fair-sampling assumption allows us to reconstruct from these data the probability *p*(*a*, *b*_0_, *b*_1_, *c*|*x*, *z*) and then to compute the quantities *I* and *J*, which appear in equation (3).

The maximum value reached in our experimental setup was 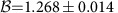, corresponding to a violation of inequality (equation (3)) of almost 20 sigmas. This value is fully compatible with a theoretical model that considers both colored and white noise in the state generated by the spontaneous parametric down-conversion process sources and takes into account the partial distinguishability of the generated photons (see Supplementary Note 2).

Next, we address the robustness of the bilocal causality inequality violation with respect to experimental noise. To this aim, we tuned the noise in the Bell-state measurement by modifying the temporal overlap between photons 2 and 3. This can be achieved by using a delay line before one of the two inputs of the BS, thus controlling the temporal delay between these photons (see Fig. 2). We can therefore define a noise parameter *p*, which is equal to 1 in the ideal case of a perfect Bell-state measurement and is equal to 0 when the probability of having a successful measurement is 1/2. This parameter can be tuned from *p*_max_ to zero by changing the delay from zero to a value larger than the coherence time of the photons.

**Figure 3:**
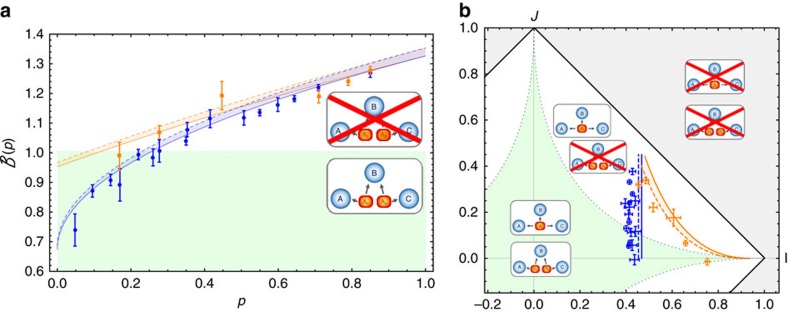
Experimental violation of bilocal causality. (**a**) Measured quantity 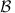 as a function of the noise parameter *p*, with fixed (blue circles) and optimized (orange squares) measurements settings. Theoretical predictions are shown by blue- and orange-shaded regions compatible with our state preparation and varying the other noise parameter *p*. The regions are obtained considering the propagation of the uncertainty in the experimental estimation of noises and are bounded by 1 s.d. upper (dashed) and lower (line) curves. The dotted horizontal line indicates the bound of the inequality (equation (3)), whereas error bars indicates 1 s.d. of uncertainty, due to Poissonian statistics. (**b**) Measured values in the *I*–*J* plane. Error bars show 1 s.d. for both *I* and *J* values. The dashed line bounds the bilocal region as prescribed by inequality (equation (3)). Green lines define the local set and the white area represents correlations, which are compatible with local models but incompatible with bilocal causality assumption. The grey area shows the set of correlations, which are incompatible with both local and bilocal models.

The measured values of 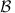 versus *p* are shown in Fig. 3a. As expected, the violation decreases with increasing noise^19^,^20^. This plot shows two sets of different data points: considering a fixed measurement basis (optimal in the absence of the additional noise) and optimizing the measurement basis at Alice and Charlie's stations as a function of *p*, that is, changing the measurement basis in order to counteract noise effects (see Supplementary Note 2). In both cases, our setup can tolerate a substantial amount of noise before inequality (equation (3)) is not violated anymore, but it is clear how the optimization increases both the degree and region of bilocal causality violation.

Another relevant way to visualize the non-bilocal correlations generated in our experiment and its relation to usual local models is displayed in Fig. 3b. A bilocal model (defined by equation (2)) must respect the inequality 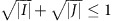, while a standard LHV model (defined by equation (1)) in turn fulfils 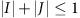. As shown in Fig. 3b, the measured values of *I* and *J* are clearly incompatible with bilocal causality (apart from the cases with the highest amount of noise) and behave in good agreement with the theoretical model. Moreover, it clearly shows how optimizing the measurement settings improves the robustness of violation against noise.

### Characterizing non-bilocal correlations against LHV models

**Figure 4:**
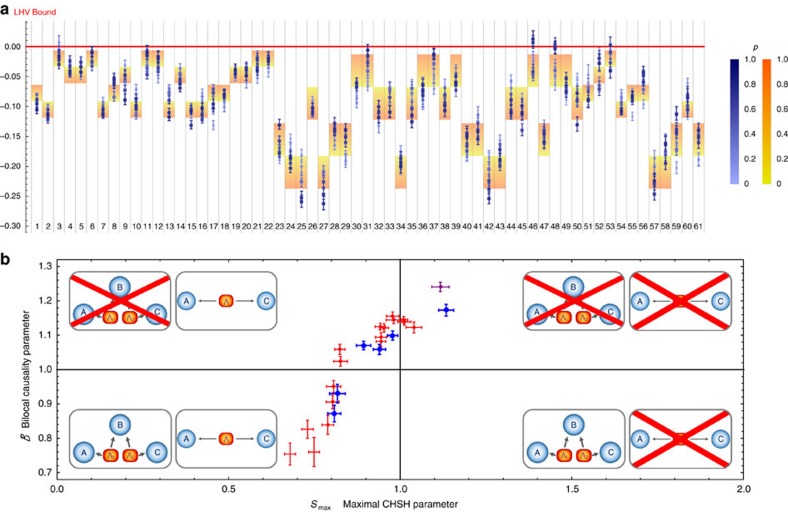
Experimental test of LHV models. (**a**) Experimental violation values for all the 61 Bell inequalities compatible with a LHV model. Each column corresponds to a different inequality, where a resulting value >0 is not compatible with an LHV model. Each point's colour represents the estimated amount of noise *p*, from dark blue (*p*=1, that is, absence of noise), to light blue (*p*=0, that is, maximum noise). Theoretical predictions are shown in the background, the red to yellow colour transition representing the dependence from *p*. Squares (circles) represent those points, which violate (do not violate) the bilocal causality inequality (equation (3)). (**b**) Comparison between experimental bilocal causality parameter and maximized CHSH parameter (multiplied by a factor 1/2 so that the local bound of the CHSH inequality is set to 1) in different regimes of noise. Bilocal causality test is performed with fixed non-optimized measurement settings, whereas CHSH maximum parameter is computed applying the Horodecki criterion (ref. 30) to a partial quantum state tomography (red points) or a complete quantum-state tomography (blue points) of the quantum state shared between A and C after the entanglement swapping protocol (that is, conditioned on singlet state outcome in station B). The purple point was evaluated directly testing both bilocal causality and CHSH in a particular regime of low noise. Circles (squares) represent entangled (separable) quantum states, where the degree of entanglement was computed via the partial transpose^46^. The lower-left region is compatible with both models, the upper-left region denotes incompatibility with bilocal causality, the upper-right region denotes violation of both the bilocal causality and CHSH inequalities, whereas the lower-right region is characterized by only CHSH violation. Error bars indicates 1 s.d. of uncertainty, due to Poissonian statistics.

The data in Fig. 3b show that the observed values for *I* and *J* do not violate the corresponding LHV inequality. However, this only represents a necessary condition. To definitively check whether we are really facing a new type of local causality violation beyond the standard LHV model (equation (1)), we also checked that all Bell inequalities defining our scenario are not violated in the experiment. In general, given an observed probability distribution, it is a simple linear program to check if it is compatible with LHV model (see, for example, ref. 27 for further details). Equivalently, noticing that a LHV model defines a polytope of correlations compatible with it^28^, one can derive all the Bell inequalities constraining that model. As described in the Supplementary Note 3, we have derived all the Bell inequalities constraining *p*(*a*, *b*_0_, *b*_1_, *c*|*x*, *z*) compatible with LHV models. Apart from trivial ones, there are 61 of these inequalities and we have checked for all the collected data with different noise parameter *p* whether they are violated. The results are shown in Fig. 4a. It can be seen that none of the points (even those that do violate the bilocal causality inequality (equation (3)), as shown in Fig. 3) show any significant evidence (taking into account the size of the error bars) for the violation of any of the all LHV constraints.

Finally, we addressed the question whether, in an entanglement swapping scenario, bilocal causality violation could represent a stronger test rather than the usual CHSH violation^29^, in order to certify non-local correlations in presence of experimental noise. We therefore performed a tomography of the quantum state shared between Alice and Charlie upon conditioning on Bob's outcome (that is, entanglement swapped state) followed by an experimental test of bilocal causality (see Supplementary Note 4). This allows us to compare our experimental bilocal causality violation with the maximum possible CHSH of the swapped state in different regimes of noise^30^. Figure 4b clearly shows the existence of quantum states, which violate bilocal causality (even without any settings' optimization) but cannot violate the CHSH inequality, thus turning unfeasible any protocol^10^,^11^,^12^ based on its violation.

## Discussion

Our results provide an experimental proof-of-principle for network generalizations of Bell's theorem. However, similarly to any Bell test^31^, our violation of the bilocal causality inequality is subjected to loopholes, in particular the locality and detection efficiency loopholes, as the parties are not space-like separated and we make use of the fair-sampling assumption. Given the nature of our experiment, a new loophole—similar to the measurement independence loophole in Bell's theorem^27^,^32^—is also introduced if the sources of states cannot be guaranteed to be truly independent. Regarding usual Bell tests, it was not until recently that such loopholes were finally overcome^2^,^3^,^4^. Thus, from the practical perspective it would be highly desirable to design future experiments achieving that also for more complex networks.

From a fundamental perspective, recent results^21^,^27^,^33^,^34^,^35^,^36^,^37^,^38^,^39^,^40^ at the interface between quantum theory and causality have shown that Bell's theorem represents a very particular case of much richer and broader range of phenomena that emerge in complex networks and that hopefully will lead to a deeper understanding of the apparent tension between quantum mechanics and our notions of causal relations. Furthermore, given the close connections between causal inference and machine learning^41^, it is pressing to consider what advantages the recent progresses in quantum machine learning^42^,^43^ can provide in such a causal context.

From a more applied perspective, such generalizations offer an almost unexplored territory and it is still unclear how to use this new form of non-local correlations in information processing. As we showed here, we can still violate a bilocal causality inequality even if the data admits a LHV model where the independence of the sources is not taken into account. That is, quantum states generating classical correlations in conventional scenarios can become powerful resources in a network, thus hopefully enlarging our current capabilities to process information in a non-classical way. For instance, a natural next step is to experimentally realize even larger quantum networks as the one shown in Fig. 1d. For sufficiently long networks, the final quantum state swapped between the end nodes may be separable and thus irrelevant as a quantum resource. Still, the correlations in the entire network might be highly non-local^25^, allowing us to probe a whole new regime in quantum information processing. Finally, we notice that during the review process of this work, an independent experimental investigation of the bilocal causality violation has appeared^44^.

## Methods

### Experimental details

Photon pairs were generated in two equal parametric down conversion sources, each one composed by a nonlinear crystal beta barium borate (BBO) injected by a pulsed pump field with *λ*=392.5 nm. The data shown in Figs 3 and 4a and the purple point in Fig. 4b were collected by using 1.5 mm -thick BBO crystals, whereas for the red and blue points in Fig. 4b we used 2 mm-thick crystals to increase the generation rate. After spectral filtering and walkoff compensation, photon are sent to the three measurement stations. The observable *A*_0_, that is, 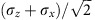, corresponds to a half-wave plate rotated by 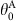
=11.25°, whereas *A*_1_, that is, 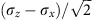, corresponds to 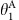
=78.75°. Analogously, *C*_0_ and *C*_1_ can be measured at Charlie's station using the same angles 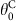
=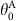 and 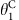
=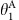.

### Data availability

The data that support the findings of this study are available from the corresponding author upon request.

## Additional information

**How to cite this article:** Carvacho, G. *et al*. Experimental violation of local causality in a quantum network. *Nat. Commun.* **8,** 14775 doi: 10.1038/ncomms14775 (2017).

**Publisher's note**: Springer Nature remains neutral with regard to jurisdictional claims in published maps and institutional affiliations.

## Supplementary Material

Supplementary Information Supplementary Figures, Supplementary Tables, Supplementary Notes and Supplementary References.
