# Supplementary material for: Experimental violation of local causality in a quantum network
Source: Nat Commun. 2017 Mar 16;8:14775. doi: 10.1038/ncomms14775 (PMC5356073; doi:10.1038/ncomms14775)
Supplement: Supplementary Information — Supplementary Figures, Supplementary Tables, Supplementary Notes and Supplementary References. [file ncomms14775-s1.pdf]

SUPPLEMENTARY NOTES 1

Experimental Details

Single photons generation was realized in two equal parametric down conversion sources, each one composed by a 1.5 mm (2 mm for red and blue points of Fig. 4-b) thick nonlinear crystal (BBO) injected by a 630 mW, 250 fs pulsed pump field with $\lambda = 392.5$ nm. The generated two photon pairs were centered at 785 nm and filtered by 3 nm interferential filters (Semrock, rectangular shaped). To compensate for the walk-off effect in the SPDC crystals between different polarizations, each photon is sent through a HWP at 45° and a 0.75 mm thick BBO crystal (1 mm for red and blue points of Fig. 4-b). Additional filtering (Semrock, long-pass edge filters) was performed to remove fluorescence and environmental noise. To compensate for equatorial phase shift between photons produced in EPR1 and EPR2 due to the propagation in fibers, photon 4 is sent through a tunable liquid crystal retarder. Photons 2 and 3 are coupled to single mode fibers, delayed one respect to the other with a controlled delay line, sent into an in-fiber 50/50 BS, and then analyzed in polarization using HWPs and PBSs. Alice's and Charlie's photons (1 and 4 in Fig. 2 of the main text) are analyzed in polarization using HWPs, whose angles identify the measurement setting, followed by PBSs. Finally, all photons are coupled to SM fibers and sent to APDs for detection. Electronic signals from APDs is analyzed through a time-to-digital-converter (id Quantique ID800). Measuring the observable A_0, *i.e.* $(\sigma_z + \sigma_x)/\sqrt{2}$, corresponds to a HWP rotated by $\theta_0^A = 11.25°$, while A_1, *i.e.* $(\sigma_z - \sigma_x)/\sqrt{2}$, corresponds to $\theta_1^A = 78.75°$. Analogously, C_0 and C_1 can be measured at Charlie's station using the same angles $\theta_0^C = \theta_0^A$ and $\theta_1^C = \theta_1^A$. By measuring fourfold coincidences for all the possible combinations (θ_i^A, θ_j^C), with $i, j = 0$ or 1, and performing the measurements with $\theta^B = 0°$ and with $\theta^B = 45°$, we are able to reconstruct the probability table to extrapolate the quantities I and J which appear in Eq. (3) of the main text.
The noise parameter p was defined as $p = 2P^B_{success} - 1$, where $P^B_{success}$ is the probability that the Bell-state measurement returns the correct outcome given an input Bell state. Since a Bell-state measurement via BS can only mix $|\psi^-\rangle$ and $|\psi^+\rangle$ (or $|\phi^-\rangle$ and $|\phi^+\rangle$) outcomes, depending on photons indistinguishability, the minimum $P^B_{success}$ will be 1/2 and it will occur in case of totally distinguishable photons. The degree of indistinguishability was computed via a Hong-Ou-Mandel dip in the fourfold coincidence counts with H (V) polarized photons in B and V (H) polarized photons in stations A and C. Indeed $P^B_{success} = [2\mathcal{C}(\infty) - \mathcal{C}(\Delta s)]/2\mathcal{C}(\infty)$ where $\mathcal{C}(\Delta s)$ is the count value at distance Δs from dip center and $\mathcal{C}(\infty)$ is the count value at the plateau. This leads to the estimation $p = [\mathcal{C}(\infty) - \mathcal{C}(\Delta s)]/\mathcal{C}(\infty)$ whose maximum experimental value was $p_{max} = 0.846 \pm 0.007$.

SUPPLEMENTARY NOTES 2

Noise Modeling

In an entanglement-swapping scenario there are different sources of noise that must be considered. The main ones affect state preparation and Bell-state measurement. Let us recall the definition of the correlation functions used in the main text given by

$$< A_x B_y C_z >= \sum_{a,b_0,b_1,c} (-1)^{a+b_y+c} p_Q(a, b_0, b_1, c|x, z). \tag{1}$$

This mean value can be evaluated with two different approaches.
The first one exploits the evaluation of the probability $p_Q(a, b_0, b_1, c|x, z)$, which reads

$$p_Q(a, b_0, b_1, c|x, z) = Tr[(P_a^x \otimes P_{b_0 b_1} \otimes P_c^z) \cdot (\varrho_{AB} \otimes \varrho_{BC})]. \tag{2}$$

where P_i denotes the projection operator on the eigenstate $|i\rangle$ and $\varrho_{AB} \otimes \varrho_{BC}$ represents the quantum state density matrix.
The second approach consists in the modeling of the quantum operators associated with equation (1), followed by a direct evaluation of the mean value through the equation:

$$< A_x B_y C_z >= Tr[(A_x \otimes B_y \otimes C_z) \cdot (\varrho_{AB} \otimes \varrho_{BC})]. \tag{3}$$

$b_0 b_1$ / y	$00(\phi^+)$	$01(\phi^-)$	$10(\psi^+)$	$11(\psi^-)$
$y=0$	1	1	-1	-1
$y=1$	1	-1	1	-1

Supplementary Figure 1: Table showing the expected values of the operator B_y, varying y, b_0 and b_1.

This second approach, which is computationally easier, allows to directly evaluate the mean values after a simple modelling of the measurement operators. It is thus possible to define the following operators

$$\begin{aligned} A_x &= (1-x)\ A_0\ +\ x\ A_1 \quad x = 0,1, \\ C_z &= (1-z)\ C_0\ +\ z\ C_1 \quad z = 0,1, \end{aligned} \tag{4}$$

where A_x and C_z are general single qubit projective measurements with eigenvalues 1 and -1.
The possible values of the operator B_y are shown in Fig. 1.
The operator B_y is then defined as

$$B_y \ = \ |\phi^+\rangle\langle\phi^+| \ + \ (1-2y)|\phi^-\rangle\langle\phi^-| \ + \ (2y-1)|\psi^+\rangle\langle\psi^+| \ - \ |\psi^-\rangle\langle\psi^-|, \tag{5}$$

which relates each value of $y = 0,\ 1$ with its correct set of outcomes.

Imperfect Bell-state measurement

Let us now derive a generalization of equation 5 for an imperfect Bell-state measurement, which we model with a well defined POVM.

Single qubit POVM

In order to introduce this model it is convenient to discuss the simpler case of a single qubit projective measurement, e.g. $\sigma_z = |0\rangle\langle 0| - |1\rangle\langle 1|$. Imperfections in the measurement can be seen as the probability $1-p$ of no success, i.e. to read $|0\rangle$ when the real state is $|1\rangle$ and viceversa. It is thus necessary to find two positive operators $\hat{F}_1$ and $\hat{F}_{-1}$ which satisfy the following constraints

$$\begin{aligned} &\sum_i \hat{F}_i = \mathbb{I} \\ &P(+1 \mid |0\rangle) \ = \ \langle 0|\ \hat{F}_{+1}\ |0\rangle \ = \ p, \\ &P(+1 \mid |1\rangle) \ = \ \langle 1|\ \hat{F}_{+1}\ |1\rangle \ = \ 1-p, \\ &P(-1 \mid |0\rangle) \ = \ \langle 0|\ \hat{F}_{-1}\ |0\rangle \ = \ 1-p, \\ &P(-1 \mid |1\rangle) \ = \ \langle 1|\ \hat{F}_{-1}\ |1\rangle \ = \ p. \end{aligned} \tag{6}$$

This can be obtained by defining the following operators

$$\begin{aligned} \hat{F}_{+1} &= p|0\rangle\langle 0| + (1-p)|1\rangle\langle 1| = \begin{pmatrix} p & 0 \\ 0 & 1-p \end{pmatrix}, \\ \hat{F}_{-1} &= (1-p)|0\rangle\langle 0| + p|1\rangle\langle 1| = \begin{pmatrix} 1-p & 0 \\ 0 & p \end{pmatrix}. \end{aligned} \tag{7}$$

Further, one can see that for an arbitrary state $|\psi\rangle = \alpha|0\rangle + \beta|1\rangle$ the following relation holds

$$\begin{aligned}
&\langle\psi|\hat{F}_{+1}|\psi\rangle = p|\alpha|^2 + (1-p)|\beta|^2 \geq 0, \\
&\langle\psi|\hat{F}_{-1}|\psi\rangle = (1-p)|\alpha|^2 + p|\beta|^2 \geq 0, \\
&\hat{F}_i = \hat{F}_i^\dagger \quad \forall i,
\end{aligned} \tag{8}$$

thus showing that these operators represent a well defined POVM.
This leads us to the definition of the following measurement operator $\hat{O}$

$$\begin{aligned}
&\hat{O}: \quad Tr[\varrho\,.\,\hat{O}] = \langle\hat{O}\rangle\,, \\
&\langle\hat{O}\rangle = \textstyle\sum_\alpha \alpha\; P(\alpha \mid \varrho) = \sum_\alpha \alpha\; Tr[\varrho\,.\,\hat{F}_\alpha] = Tr[\varrho\,.\,(\sum_\alpha \alpha\hat{F}_\alpha)] \equiv Tr[\varrho\,.\,\hat{O}], \\
&\hat{O} \equiv \textstyle\sum_\alpha \alpha\hat{F}_\alpha = \hat{F}_{+1} - \hat{F}_{-1},
\end{aligned} \tag{9}$$

which models an imperfect measurement.

POVM generalization of B_y

A Bell-state measurement in a Beam Splitter (BS) strongly relies on the indistinguishability of the photons. It has been shown [1] that partial distinguishability in Bell-state measurement acts mixing the $|\psi^+\rangle$ results with $|\psi^-\rangle$ ones and viceversa (and similarly for $|\phi^+\rangle$ and $|\phi^-\rangle$ states), but it doesn't mix elements belonging to these two different categories. In this section we will develop the single qubit case shown before, extending it to the two-qubit case, and taking into account that an imperfect Bell-state measurement can only mix $|\phi^+\rangle$ with $|\phi^-\rangle$ or $|\psi^+\rangle$ with $|\psi^-\rangle$.
Following the previous conclusions we can rewrite equation (5) as

$$\begin{aligned}
&a_1 \equiv 1,\; a_2 \equiv 1-2y,\; a_3 \equiv 2y-1,\; a_4 \equiv -1, \\
&B_y \;=\; a_1|\phi^+\rangle\langle\phi^+| \;+\; a_2|\phi^-\rangle\langle\phi^-| \;+\; a_3|\psi^+\rangle\langle\psi^+| \;+\; a_4|\psi^-\rangle\langle\psi^-|.
\end{aligned} \tag{10}$$

And then apply the substitution

$$\begin{aligned}
|\phi^+\rangle\langle\phi^+| \to \hat{F}_1 = \frac{1+p}{2}|\phi^+\rangle\langle\phi^+| + \frac{1-p}{2}|\phi^-\rangle\langle\phi^-|, \\
|\phi^-\rangle\langle\phi^-| \to \hat{F}_2 = \frac{1+p}{2}|\phi^-\rangle\langle\phi^-| + \frac{1-p}{2}|\phi^+\rangle\langle\phi^+|, \\
|\psi^+\rangle\langle\psi^+| \to \hat{F}_3 = \frac{1+p}{2}|\psi^+\rangle\langle\psi^+| + \frac{1-p}{2}|\psi^-\rangle\langle\psi^-|, \\
|\psi^-\rangle\langle\psi^-| \to \hat{F}_4 = \frac{1+p}{2}|\psi^-\rangle\langle\psi^-| + \frac{1-p}{2}|\psi^+\rangle\langle\psi^+|,
\end{aligned} \tag{11}$$

where p was chosen as a parameter of imperfection such that the probability of success is never below 50% for $0 \leq p \leq 1$.

Equation (11) gives the correct probabilities

$$
\begin{aligned}
P(a_1 \mid |\phi^+\rangle) &= \frac{1+p}{2} \;\; , \;\; P(a_2 \mid |\phi^+\rangle) = \frac{1-p}{2}, \\
P(a_1 \mid |\phi^-\rangle) &= \frac{1-p}{2} \;\; , \;\; P(a_2 \mid |\phi^-\rangle) = \frac{1+p}{2}, \\
P(a_3 \mid |\psi^+\rangle) &= \frac{1+p}{2} \;\; , \;\; P(a_4 \mid |\psi^+\rangle) = \frac{1-p}{2}, \\
P(a_3 \mid |\psi^-\rangle) &= \frac{1-p}{2} \;\; , \;\; P(a_4 \mid |\psi^-\rangle) = \frac{1+p}{2},
\end{aligned} \tag{12}
$$

and at the same time it satisfies that

$$
\begin{aligned}
&\textstyle\sum_i \hat{F}_i = \mathbb{I} \;\; , \;\; \hat{F}_i = \hat{F}_i^\dagger \;\; \forall i, \\
&\langle\psi|\hat{F}_1|\psi\rangle = \frac{1+p}{2}|\alpha|^2 + \frac{1-p}{2}|\beta|^2 \geq 0, \\
&\langle\psi|\hat{F}_2|\psi\rangle = \frac{1-p}{2}|\alpha|^2 + \frac{1+p}{2}|\beta|^2 \geq 0, \\
&\langle\psi|\hat{F}_3|\psi\rangle = \frac{1+p}{2}|\gamma|^2 + \frac{1-p}{2}|\delta|^2 \geq 0, \\
&\langle\psi|\hat{F}_4|\psi\rangle = \frac{1-p}{2}|\gamma|^2 + \frac{1+p}{2}|\delta|^2 \geq 0,
\end{aligned} \tag{13}
$$

for an arbitrary state

$$
|\psi\rangle = \alpha|\phi^+\rangle + \beta|\phi^-\rangle + \gamma|\psi^+\rangle + \delta|\psi^-\rangle.
$$

These constraints guarantee that F_i operators shown in equation (11) represent a well defined POVM. The imperfect Bell-state measurement is then modeled as

$$
B_y \;=\; a_1\,\hat{F}_1 \;+\; a_2\,\hat{F}_2 \;+\; a_3\,\hat{F}_3 \;+\; a_4\,\hat{F}_4. \tag{14}
$$

Presence of noise in the quantum state preparation

In our entanglement swapping scenario two pairs of entangled photons in the singlet state are generated by two independent SPDC sources. It has been shown [2] that these sources suffer from two different kinds of noise:

1) White noise (i.e. isotropic depolarization):

$$
\varrho = \; v\,|\psi^-\rangle\langle\psi^-| \;+\; (1-v)\,\mathbb{I}/4, \tag{15}
$$

where $\mathbb{I}$ represents the identity matrix.

2) Colored noise (i.e. depolarization on a preferred direction):

$$
\varrho = \; v\,|\psi^-\rangle\langle\psi^-| \;+\; \frac{(1-v)}{2}\begin{pmatrix} 0&0&0&0\\ 0&1&0&0\\ 0&0&1&0\\ 0&0&0&0 \end{pmatrix} = \; v\,|\psi^-\rangle\langle\psi^-| \;+\; \frac{(1-v)}{2}(|\psi^-\rangle\langle\psi^-| + |\psi^+\rangle\langle\psi^+|). \tag{16}
$$

The state given by this mixture of noises can be modeled as:

$$
\varrho_{XB} = \; v_X\,|\psi^-\rangle\langle\psi^-| \;+\; (1-v_X)\cdot[\lambda_X\frac{|\psi^-\rangle\langle\psi^-| + |\psi^+\rangle\langle\psi^+|}{2} + (1-\lambda_X)\frac{\mathbb{I}}{4}], \tag{17}
$$

where v_X stands for the total noise while λ_X is the fraction of colored noise and where the label X can represent either the measurement station A or the measurement station C.

Violation of bilocal causality depending on noise

The presence of noise in the experimental setup tends to destroy quantum correlations, reducing QM's Bell non-local behaviour. It is thus essential to estimate the dependence of the parameter $\mathcal{B}$ (defining the bilocality inequality) from experimental noise in our model. This can be achieved using equations (14) and (17) and applying equation (3).
Let Alice and Charlie perform the following measurements (A_0, A_1 and C_0, C_1 respectively) :

$$\begin{aligned} A_0 &= cos(\theta)\sigma_z + sin(\theta)\sigma_x, \quad A_1 = cos(\theta)\sigma_z - sin(\theta)\sigma_x, \\ C_0 &= cos(\phi)\sigma_z + sin(\phi)\sigma_x, \quad C_1 = cos(\phi)\sigma_z - sin(\phi)\sigma_x. \end{aligned} \tag{18}$$

After several algebraic manipulations we obtain

$$\begin{aligned} &\langle A_x B_y C_z \rangle = -\beta(v_A, v_C, \lambda_A, \lambda_C) \cdot (-1+y) cos(\theta) cos(\phi) + \alpha(v_A, v_C, p) \cdot (-1+2x) y (-1+2z)\; sin(\theta) sin(\phi), \\ &x = 0,1 \quad y = 0,1 \quad z = 0,1, \end{aligned} \tag{19}$$

where we defined

$$\begin{aligned} \alpha(v_A, v_C, p) &= p\, v_A\, v_C, \\ \beta(v_A, v_C, \lambda_A, \lambda_C) &= (-v_A(\lambda_A - 1) + \lambda_A) \cdot (-v_C(\lambda_C - 1) + \lambda_C). \end{aligned} \tag{20}$$

Hereafter we will restrict to the case of interest given by

$$\begin{aligned} v_A &= v_C = \sqrt{v}, \\ \lambda_A &= \lambda_C = \lambda. \end{aligned} \tag{21}$$

Thus, equation (19) leads to

$$\mathcal{B}(\alpha, \beta, \theta, \phi) = \sqrt{|\beta(v, \lambda) \cdot cos(\theta) cos(\phi)|} + \sqrt{|\alpha(v, p) \cdot sin(\theta) sin(\phi)|}. \tag{22}$$

In the following to analyze the maximal value of $\mathcal{B}$ as a function of noise. This can be done by considering α and β as parameters and then maximizing the function $\mathcal{B}(\theta, \phi)$. This leads to the equations

$$\begin{aligned} \frac{\partial \mathcal{B}(\theta,\phi)}{\partial \theta} &= \frac{1}{2}\sqrt{\alpha |sin(\theta) sin(\phi)|}\; cot(\theta) - \frac{1}{2}\sqrt{\beta |cos(\theta) cos(\phi)|}\; tan(\theta) = 0, \\ \frac{\partial \mathcal{B}(\theta,\phi)}{\partial \phi} &= \frac{1}{2}\sqrt{\alpha |sin(\theta) sin(\phi)|}\; cot(\phi) - \frac{1}{2}\sqrt{\beta |cos(\theta) cos(\phi)|}\; tan(\phi) = 0. \end{aligned} \tag{23}$$

These system of equations admits only solutions constrained by

$$tan(\theta)^2 = tan(\phi)^2 \;\; \leftrightarrow \;\; \phi = \pm\theta + n\pi \;,\; n \in \mathbb{Z}. \tag{24}$$

The maximization of equation (22) is thus equivalent to maximize the simplified expression

$$\mathcal{B}(\theta) = \sqrt{\beta} \cdot |\; cos[\theta(\alpha, \beta)] \;| + \sqrt{\alpha} \cdot |\; sin[\theta(\alpha, \beta)] \;|, \tag{25}$$

that leads to

$$\begin{aligned} &\theta(\alpha,\ \beta) = ArcTan(\sqrt{\frac{\alpha(v,p)}{\beta(v,\lambda)}}), \\ &\phi(\alpha,\ \beta) = \pm\theta(\alpha,\ \beta) + n\pi \;,\; n \in \mathbb{Z}, \\ &\mathcal{B}_{max}(\alpha(v,p), \beta(v,\lambda)) = \sqrt{\alpha(v,p) + \beta(v,\lambda)}. \end{aligned} \tag{26}$$

Importantly, these analytical results allow for the evaluation of the best experimental settings (i.e. $\theta(\alpha,\beta)$) given particular experimental conditions (i.e. α and β).
It is now interesting to study how equation (26) behaves in different regimes of noise. When dealing with colored noise only (i.e. $\lambda = 1$), so that $\beta(v, \lambda = 1) = 1$, it is possible to perform a full optimization, allowing for bilocality violation for all possible values of v and p.
In the opposite case (i.e. $\lambda = 0$, white noise only) we have $\beta(v, \lambda = 0) = v^2$. The noise parameter v can be factorized while the dependence of $\theta(\alpha,\beta)$ from v vanishes. This leads to the conclusion that no settings optimization can counteract the effects of white noise in the state preparation. However, performing the optimized measurements settings prescribed in equation (26) allows also to maximize $\mathcal{B}$ with respect to the imperfection in the Bell-state measurement due to partial distinguishability in the BS (i.e. parameter p), which means that an advantage can always be obtained by such optimization.

Violation of bilocal causality for a wider class of measurements

We now extend the conclusions of the previous section to the case in which Alice and Charlie can perform a wider class of measurements. First, let us define

$$M(\zeta,\eta) = sin(\eta)sin(\zeta)\sigma_y + cos(\eta)sin(\zeta)\sigma_x + cos(\zeta)\sigma_z. \tag{27}$$

Suppose A and C perform the following measurements:

$$\begin{aligned} A_0 &= M(\theta_0,\gamma_0), \quad & A_1 &= M(\theta_1,\gamma_1), \\ C_0 &= M(\phi_0,\delta_0), \quad & C_1 &= M(\phi_1,\delta_1). \end{aligned} \tag{28}$$

This choice leads to

$$\begin{aligned} \mathcal{B}(\alpha,\beta) &= \frac{1}{2}\{\sqrt{|\beta|\cdot|cos(\theta_0)+cos(\theta_1)|}\cdot\sqrt{|cos(\phi_0)+cos(\phi_1)|} \\ &+\sqrt{|\alpha|\cdot|cos(\gamma_0)sin(\theta_0)-cos(\gamma_1)sin(\theta_1)|}\cdot\sqrt{|cos(\delta_0)sin(\phi_0)-cos(\delta_1)sin(\phi_1)|}\}. \end{aligned} \tag{29}$$

Further, it is possible to show that

$$\begin{aligned} \mathcal{B}(\alpha,\beta) &\leq \frac{1}{2}\{\sqrt{|\beta|\cdot|cos(\theta_0)+cos(\theta_1)|}\cdot\sqrt{|cos(\phi_0)+cos(\phi_1)|} \\ &+\sqrt{|\alpha|\cdot[|sin(\theta_0)|+|sin(\theta_1)|]}\cdot\sqrt{[|sin(\phi_0)|+|sin(\phi_1)|]}\ \} \equiv \tilde{\mathcal{B}}(\theta_0,\theta_1,\phi_0,\phi_1). \end{aligned} \tag{30}$$

In order to maximize $\tilde{\mathcal{B}}(\theta_0,\theta_1,\phi_0,\phi_1)$, it is necessary to find its stationary points, solving the equations:

$$\begin{aligned} \frac{\partial\tilde{\mathcal{B}}(\theta_0,\theta_1,\phi_0,\phi_1)}{\partial\theta_0} &= \Gamma(\phi_0,\phi_1,\theta_0,\theta_1)cos(\theta_0)\cdot sign[sin(\theta_0)] - \Omega(\phi_0,\phi_1,\theta_0,\theta_1)sin(\theta_0) = 0, \\ \frac{\partial\tilde{\mathcal{B}}(\theta_0,\theta_1,\phi_0,\phi_1)}{\partial\theta_1} &= \Gamma(\phi_0,\phi_1,\theta_0,\theta_1)cos(\theta_1)\cdot sign[sin(\theta_1)] - \Omega(\phi_0,\phi_1,\theta_0,\theta_1)sin(\theta_1) = 0, \\ \frac{\partial\tilde{\mathcal{B}}(\theta_0,\theta_1,\phi_0,\phi_1)}{\partial\phi_0} &= \Gamma(\theta_0,\theta_1,\phi_0,\phi_1)cos(\phi_0)\cdot sign[sin(\phi_0)] - \Omega(\theta_0,\theta_1,\phi_0,\phi_1)sin(\phi_0) = 0, \\ \frac{\partial\tilde{\mathcal{B}}(\theta_0,\theta_1,\phi_0,\phi_1)}{\partial\phi_1} &= \Gamma(\theta_0,\theta_1,\phi_0,\phi_1)cos(\phi_1)\cdot sign[sin(\phi_1)] - \Omega(\theta_0,\theta_1,\phi_0,\phi_1)sin(\phi_1) = 0, \end{aligned} \tag{31}$$

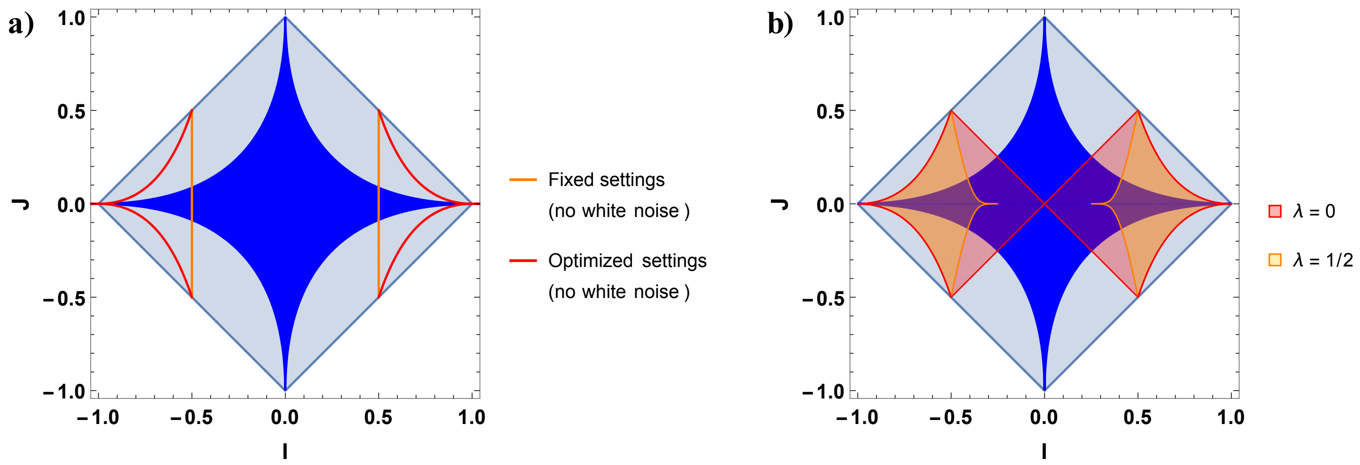

Supplementary Figure 2: QM behaviour in the I, J plane. Light blue region represents the local set while dark blue region shows the bilocal set. **(a)** QM behaviour in the case of absence of white noise ($\lambda = 1$ or $v = 1$). Orange line shows QM behaviour when A, C perform the measurements given in equation (18) with fixed values $\theta = \phi = \pi/4$, while red line shows its behaviour with the optimized settings described in (26). **(b)** Region allowed to QM for different values of v, p when dealing with optimized settings. Red and orange regions shows the cases when $\lambda = 0,\ 1/2$ respectively.

where we have defined

$$\Gamma(\zeta,\eta,\nu,\mu) = \frac{1}{4}\sqrt{\frac{\alpha\cdot(|sin(\zeta)|+|sin(\eta)|)}{|sin(\nu)|+|sin(\mu)|}},$$
$$\Omega(\zeta,\eta,\nu,\mu) = \frac{1}{4}\sqrt{\frac{\beta\cdot|cos(\zeta)+cos(\eta)|}{|cos(\nu)+cos(\mu)|}}\cdot sign[cos(\zeta)+cos(\eta)]. \tag{32}$$

Excluding singular points, the solutions of the system (31) are constrained by:

$$\frac{|sin(\theta_0)|}{cos(\theta_0)} = \frac{|sin(\theta_1)|}{cos(\theta_1)} \longrightarrow \theta_1 = \pm\theta_0 + 2\pi n,\ \ n\in\mathbb{Z},$$
$$\frac{|sin(\phi_0)|}{cos(\phi_0)} = \frac{|sin(\phi_1)|}{cos(\phi_1)} \longrightarrow \phi_1 = \pm\phi_0 + 2\pi n,\ \ n\in\mathbb{Z}. \tag{33}$$

The problem of finding stationary points for $\tilde{\mathcal{B}}(\theta_0,\theta_1,\phi_0,\phi_1)$ can thus be simplified, leading to

$$\max_{\theta_0,\theta_1,\phi_0,\phi_1}[\tilde{\mathcal{B}}(\theta_0,\theta_1,\phi_0,\phi_1)] = \max_{\theta_0,\phi_0}[\sqrt{\beta\cdot|cos(\theta_0)|}\cdot\sqrt{|cos(\phi_0)|}+\sqrt{\alpha\cdot|sin(\theta_0)sin(\phi_0)|}\,]. \tag{34}$$

This demonstrates that equation (22) maximizes equation (29). We conclude then that the measurements defined in equation (18) represent the subset of the class given in equation (28) which provides a full optimization of the parameter $\mathcal{B}$. Numerical maximization of equation (29) was also performed and numerical results perfectly agree with these analytical conclusions.

Local and bilocal correlations

We focus now on the relation between QM and both the local and the bilocal set. As discussed in the main text, in terms of the observables I and J the following inequalities hold

$$\textit{Local set}: \quad \mathcal{L} \equiv |I| + |J| \leq 1, \tag{35}$$

$$\textit{Bilocal set}: \quad \mathcal{B} \equiv \sqrt{|I|} + \sqrt{|J|} \leq 1. \tag{36}$$

QM mean values $\langle I \rangle$ and $\langle J \rangle$ depend on several factors, such as the measurements performed in A and C, the quantum state preparation an the noise level under consideration in the Bell-state measurement. Considering our noise modeling and performing the optimized measurements described in equation (26) it is possible to obtain

$$\begin{aligned} \langle I \rangle &= \pm \frac{\beta(v,\lambda)^2}{\beta(v,\lambda)+\alpha(v,p)}, \\ \langle J \rangle &= \pm \frac{\alpha(v,p)^2}{\beta(v,\lambda)+\alpha(v,p)}, \end{aligned} \tag{37}$$

where the sign $\pm$ depends on the choice of $\phi(\alpha,\beta) = n\pi \pm \theta(\alpha,\beta)$, $n \in \mathbb{Z}$. For the case $v = 1$ (regardless of λ) or $\lambda = 1$ (regardless of v) equation (37) leads to the QM values shown in Fig. 2-1 (red line).
It is interesting to compare this result with the orange line, which shows QM behaviour in case of fixed measurement angles ($\theta = \pi/4$ and $\phi = n\pi \pm \pi/4$, $n \in \mathbb{Z}$). It is clear that the maximization protocol keeps QM out of the bilocal set. In the presence of white noise, after a certain threshold value, measurement optimization is not able to keep QM completely out of the bilocal set (see Fig. 2-2). It is worth pointing out that equation (37) never allows QM to exit the local region, regardless of the value of v, p, and λ. As a matter of fact, no violation of the inequality $|I| + |J| \leq 1$ is possible within quantum mechanics thought some non-signaling (post-quantum) correlations can do so [3].

SUPPLEMENTARY NOTES 3

Comprehensive Bell inequalities test

We tested our data using all the Bell inequalities implied by LHV models. To that aim, notice that a LHV model implies that any distribution compatible with it lies inside a convex set known as the correlation polytope [6]. In our case we have to characterize the polytope associated with the probability distribution $p(a, b_0, b_1, c|x, z)$. This can be done by simply listing all the classical deterministic strategies, for example, $p(a, b_0, b_1, c|x, z) = \delta_{a,1}\delta_{b_0,1}\delta_{b_1,1}\delta_{c,1}$ (that is, the probability is equal to one if all the outputs are $+1$ and zero otherwise). There are 64 of these deterministic strategies. We have dualized the description using standard convex optimization software [7] and obtained all the corresponding constraints. Some of these are equality constraints, more specifically the normalization and non-signaling conditions. Among the inequalities, there are many simple positivity constraints. Finally, the output of our computation includes other 61 inequalities (tested in Fig. 4-a of the main text) which are listed below.

$\mathcal{I}_1 = -p(1,1,1,0|1,1) + p(1,1,1,1|1,0) - p(1,1,1,1|1,1) \leq 0$
$\mathcal{I}_2 = -p(1,1,0,0|1,1) + p(1,1,0,1|1,0) - p(1,1,0,1|1,1) \leq 0$
$\mathcal{I}_3 = -p(1,0,1,0|1,1) + p(1,0,1,1|1,0) - p(1,0,1,1|1,1) \leq 0$
$\mathcal{I}_4 = -p(1,0,0,0|1,1) + p(1,0,0,1|1,0) - p(1,0,0,1|1,1) \leq 0$
$\mathcal{I}_5 = -p(0,1,1,0|1,1) + p(0,1,1,1|1,0) - p(0,1,1,1|1,1) \leq 0$
$\mathcal{I}_6 = -p(0,1,0,0|1,1) + p(0,1,0,1|1,0) - p(0,1,0,1|1,1) \leq 0$
$\mathcal{I}_7 = -p(0,0,1,0|1,1) + p(0,0,1,1|1,0) - p(0,0,1,1|1,1) \leq 0$
$\mathcal{I}_8 = -p(0,1,1,1|1,1) + p(1,1,1,1|0,1) - p(1,1,1,1|1,1) \leq 0$
$\mathcal{I}_9 = -p(0,1,1,0|1,1) + p(1,1,1,0|0,1) - p(1,1,1,0|1,1) \leq 0$
$\mathcal{I}_{10} = -p(0,1,0,1|1,1) + p(1,1,0,1|0,1) - p(1,1,0,1|1,1) \leq 0$
$\mathcal{I}_{11} = -p(0,1,0,0|1,1) + p(1,1,0,0|0,1) - p(1,1,0,0|1,1) \leq 0$
$\mathcal{I}_{12} = -p(0,0,1,1|1,1) + p(1,0,1,1|0,1) - p(1,0,1,1|1,1) \leq 0$
$\mathcal{I}_{13} = -p(0,0,1,0|1,1) + p(1,0,1,0|0,1) - p(1,0,1,0|1,1) \leq 0$
$\mathcal{I}_{14} = -p(0,0,0,1|1,1) + p(1,0,0,1|0,1) - p(1,0,0,1|1,1) \leq 0$
$\mathcal{I}_{15} = -p(1,1,1,0|0,1) + p(1,1,1,1|0,0) - p(1,1,1,1|0,1) \leq 0$
$\mathcal{I}_{16} = -p(0,1,1,1|1,0) + p(1,1,1,1|0,0) - p(1,1,1,1|1,0) \leq 0$
$\mathcal{I}_{17} = -p(1,1,0,0|0,1) + p(1,1,0,1|0,0) - p(1,1,0,1|0,1) \leq 0$
$\mathcal{I}_{18} = -p(0,1,0,1|1,0) + p(1,1,0,1|0,0) - p(1,1,0,1|1,0) \leq 0$
$\mathcal{I}_{19} = -p(1,0,1,0|0,1) + p(1,0,1,1|0,0) - p(1,0,1,1|0,1) \leq 0$
$\mathcal{I}_{20} = -p(0,0,1,1|1,0) + p(1,0,1,1|0,0) - p(1,0,1,1|1,0) \leq 0$
$\mathcal{I}_{21} = -p(1,0,0,0|0,1) + p(1,0,0,1|0,0) - p(1,0,0,1|0,1) \leq 0$
$\mathcal{I}_{22} = -p(0,0,0,1|1,0) + p(1,0,0,1|0,0) - p(1,0,0,1|1,0) \leq 0$
$\mathcal{I}_{23} = -p(0,1,1,1|1,0) - p(1,1,1,0|0,1) + p(1,1,1,1|0,0) - p(1,1,1,1|1,1) \leq 0$
$\mathcal{I}_{24} = -p(0,1,1,1|1,1) - p(1,1,1,0|0,1) + p(1,1,1,1|0,0) - p(1,1,1,1|1,0) \leq 0$
$\mathcal{I}_{25} = -p(0,1,1,1|1,0) - p(1,1,1,0|1,1) + p(1,1,1,1|0,0) - p(1,1,1,1|0,1) \leq 0$
$\mathcal{I}_{26} = -p(0,1,1,0|1,1) + p(1,1,1,1|0,0) - p(1,1,1,1|0,1) - p(1,1,1,1|1,0) \leq 0$
$\mathcal{I}_{27} = -p(0,1,0,1|1,0) - p(1,1,0,0|0,1) + p(1,1,0,1|0,0) - p(1,1,0,1|1,1) \leq 0$
$\mathcal{I}_{28} = -p(0,1,0,1|1,1) - p(1,1,0,0|0,1) + p(1,1,0,1|0,0) - p(1,1,0,1|1,0) \leq 0$
$\mathcal{I}_{29} = -p(0,1,0,1|1,0) - p(1,1,0,0|1,1) + p(1,1,0,1|0,0) - p(1,1,0,1|0,1) \leq 0$
$\mathcal{I}_{30} = -p(0,1,0,0|1,1) + p(1,1,0,1|0,0) - p(1,1,0,1|0,1) - p(1,1,0,1|1,0) \leq 0$
$\mathcal{I}_{31} = -p(0,0,1,1|1,0) - p(1,0,1,0|0,1) + p(1,0,1,1|0,0) - p(1,0,1,1|1,1) \leq 0$
$\mathcal{I}_{32} = -p(0,0,1,1|1,1) - p(1,0,1,0|0,1) + p(1,0,1,1|0,0) - p(1,0,1,1|1,0) \leq 0$
$\mathcal{I}_{33} = -p(0,0,1,1|1,0) - p(1,0,1,0|1,1) + p(1,0,1,1|0,0) - p(1,0,1,1|0,1) \leq 0$
$\mathcal{I}_{34} = -p(0,0,1,0|1,1) + p(1,0,1,1|0,0) - p(1,0,1,1|0,1) - p(1,0,1,1|1,0) \leq 0$
$\mathcal{I}_{35} = -p(0,0,0,1|1,0) - p(1,0,0,0|0,1) + p(1,0,0,1|0,0) - p(1,0,0,1|1,1) \leq 0$
$\mathcal{I}_{36} = -p(0,0,0,1|1,1) - p(1,0,0,0|0,1) + p(1,0,0,1|0,0) - p(1,0,0,1|1,0) \leq 0$
$\mathcal{I}_{37} = -p(0,0,0,1|1,0) - p(1,0,0,0|1,1) + p(1,0,0,1|0,0) - p(1,0,0,1|0,1) \leq 0$
$\mathcal{I}_{38} = -p(0,0,0,1|1,1) - p(1,0,0,0|1,1) - p(1,0,0,1|0,0) + p(1,0,0,1|0,1) + p(1,0,0,1|1,0) - p(1,0,0,1|1,1) \leq 0$
$\mathcal{I}_{39} = -p(0,0,1,1|1,1) - p(1,0,1,0|1,1) - p(1,0,1,1|0,0) + p(1,0,1,1|0,1) + p(1,0,1,1|1,0) - p(1,0,1,1|1,1) \leq 0$

Continues in the next page.

$$\mathcal{I}_{40} = -p(0,0,1,0|1,1) + p(0,0,1,1|1,0) - p(0,0,1,1|1,1) - p(1,0,1,1|0,0) + p(1,0,1,1|0,1) - p(1,0,1,1|1,1) \leq 0$$
$$\mathcal{I}_{41} = -p(0,0,1,0|1,1) + p(1,0,1,0|0,1) - p(1,0,1,0|1,1) - p(1,0,1,1|0,0) + p(1,0,1,1|1,0) - p(1,0,1,1|1,1) \leq 0$$
$$\mathcal{I}_{42} = -p(0,0,1,0|1,1) + p(0,0,1,1|1,0) - p(0,0,1,1|1,1) + p(1,0,1,0|0,1) - p(1,0,1,0|1,1) - p(1,0,1,1|0,0) \leq 0$$
$$\mathcal{I}_{43} = -p(0,1,0,1|1,1) - p(1,1,0,0|1,1) - p(1,1,0,1|0,0) + p(1,1,0,1|0,1) + p(1,1,0,1|1,0) - p(1,1,0,1|1,1) \leq 0$$
$$\mathcal{I}_{44} = -p(0,1,0,0|1,1) + p(0,1,0,1|1,0) - p(0,1,0,1|1,1) - p(1,1,0,1|0,0) + p(1,1,0,1|0,1) - p(1,1,0,1|1,1) \leq 0$$
$$\mathcal{I}_{45} = -p(0,1,0,0|1,1) + p(1,1,0,0|0,1) - p(1,1,0,0|1,1) - p(1,1,0,1|0,0) + p(1,1,0,1|1,0) - p(1,1,0,1|1,1) \leq 0$$
$$\mathcal{I}_{46} = -p(0,1,0,0|1,1) + p(0,1,0,1|1,0) - p(0,1,0,1|1,1) + p(1,1,0,0|0,1) - p(1,1,0,0|1,1) - p(1,1,0,1|0,0) \leq 0$$
$$\mathcal{I}_{47} = -p(0,1,1,1|1,1) - p(1,1,1,0|1,1) - p(1,1,1,1|0,0) + p(1,1,1,1|0,1) + p(1,1,1,1|1,0) - p(1,1,1,1|1,1) \leq 0$$
$$\mathcal{I}_{48} = -p(0,1,1,0|1,1) + p(0,1,1,1|1,0) - p(0,1,1,1|1,1) - p(1,1,1,1|0,0) + p(1,1,1,1|0,1) - p(1,1,1,1|1,1) \leq 0$$
$$\mathcal{I}_{49} = -p(0,1,1,0|1,1) + p(1,1,1,0|0,1) - p(1,1,1,0|1,1) - p(1,1,1,1|0,0) + p(1,1,1,1|1,0) - p(1,1,1,1|1,1) \leq 0$$
$$\mathcal{I}_{50} = -p(0,1,1,0|1,1) + p(0,1,1,1|1,0) - p(0,1,1,1|1,1) + p(1,1,1,0|0,1) - p(1,1,1,0|1,1) - p(1,1,1,1|0,0) \leq 0$$
$$\mathcal{I}_{51} = -p(0,0,1,0|1,1) + p(0,0,1,1|1,0) - p(0,0,1,1|1,1) + p(1,0,1,0|0,1) - p(1,0,1,0|1,1) - p(1,0,1,1|0,0)$$
$$+p(1,0,1,1|0,1) + p(1,0,1,1|1,0) - p(1,0,1,1|1,1) \leq 0$$
$$\mathcal{I}_{52} = -p(0,1,0,0|1,1) + p(0,1,0,1|1,0) - p(0,1,0,1|1,1) + p(1,1,0,0|0,1) - p(1,1,0,0|1,1) - p(1,1,0,1|0,0)$$
$$+p(1,1,0,1|0,1) + p(1,1,0,1|1,0) - p(1,1,0,1|1,1) \leq 0$$
$$\mathcal{I}_{53} = -p(0,1,1,0|1,1) + p(0,1,1,1|1,0) - p(0,1,1,1|1,1) + p(1,1,1,0|0,1) - p(1,1,1,0|1,1) - p(1,1,1,1|0,0)$$
$$+p(1,1,1,1|0,1) + p(1,1,1,1|1,0) - p(1,1,1,1|1,1) \leq 0$$
$$\mathcal{I}_{54} = -1 + p(0,0,0,1|1,1) + p(0,0,1,0|1,1) + p(0,0,1,1|1,1) + p(0,1,0,0|1,1) + p(0,1,0,1|1,1) + p(0,1,1,0|1,1)$$
$$+p(0,1,1,1|1,1) + p(1,0,0,0|1,1) + p(1,0,0,1|1,1) + p(1,0,1,0|1,1) + p(1,0,1,1|1,1) + p(1,1,0,0|1,1)$$
$$+p(1,1,0,1|1,1) + p(1,1,1,0|1,1) + p(1,1,1,1|1,1) \leq 0$$
$$\mathcal{I}_{55} = -1 + p(0,0,0,1|1,0) + p(0,0,1,0|1,1) + p(0,0,1,1|1,1) + p(0,1,0,0|1,1) + p(0,1,0,1|1,1) + p(0,1,1,0|1,1)$$
$$+p(0,1,1,1|1,1) + p(1,0,0,0|1,1) + p(1,0,0,1|1,1) + p(1,0,1,0|1,1) + p(1,0,1,1|1,1) + p(1,1,0,0|1,1)$$
$$+p(1,1,0,1|1,1) + p(1,1,1,0|1,1) + p(1,1,1,1|1,1) \leq 0$$
$$\mathcal{I}_{56} = -1 + p(0,0,0,1|1,1) + p(0,0,1,0|1,1) + p(0,0,1,1|1,1) + p(0,1,0,0|1,1) + p(0,1,0,1|1,1) + p(0,1,1,0|1,1)$$
$$+p(0,1,1,1|1,1) + p(1,0,0,0|0,1) + p(1,0,0,1|1,1) + p(1,0,1,0|1,1) + p(1,0,1,1|1,1) + p(1,1,0,0|1,1)$$
$$+p(1,1,0,1|1,1) + p(1,1,1,0|1,1) + p(1,1,1,1|1,1) \leq 0$$
$$\mathcal{I}_{57} = -1 + p(0,0,0,1|1,0) + p(0,0,1,0|1,1) + p(0,0,1,1|1,1) + p(0,1,0,0|1,1) + p(0,1,0,1|1,1) + p(0,1,1,0|1,1)$$
$$+p(0,1,1,1|1,1) + p(1,0,0,0|1,1) - p(1,0,0,1|0,0) + p(1,0,0,1|0,1) + p(1,0,1,0|1,1) + p(1,0,1,1|1,1)$$
$$+p(1,1,0,0|1,1) + p(1,1,0,1|1,1) + p(1,1,1,0|1,1) + p(1,1,1,1|1,1) \leq 0$$
$$\mathcal{I}_{58} = -1 + p(0,0,0,1|1,1) + p(0,0,1,0|1,1) + p(0,0,1,1|1,1) + p(0,1,0,0|1,1) + p(0,1,0,1|1,1) + p(0,1,1,0|1,1)$$
$$+p(0,1,1,1|1,1) + p(1,0,0,0|0,1) - p(1,0,0,1|0,0) + p(1,0,0,1|1,0) + p(1,0,1,0|1,1) + p(1,0,1,1|1,1)$$
$$+p(1,1,0,0|1,1) + p(1,1,0,1|1,1) + p(1,1,1,0|1,1) + p(1,1,1,1|1,1) \leq 0$$
$$\mathcal{I}_{59} = -1 + p(0,0,0,1|1,0) + p(0,0,1,0|1,1) + p(0,0,1,1|1,1) + p(0,1,0,0|1,1) + p(0,1,0,1|1,1) + p(0,1,1,0|1,1)$$
$$+p(0,1,1,1|1,1) + p(1,0,0,0|0,1) - p(1,0,0,1|0,0) + p(1,0,0,1|1,1) + p(1,0,1,0|1,1) + p(1,0,1,1|1,1)$$
$$+p(1,1,0,0|1,1) + p(1,1,0,1|1,1) + p(1,1,1,0|1,1) + p(1,1,1,1|1,1) \leq 0$$
$$\mathcal{I}_{60} = -1 + p(0,0,0,1|1,0) + p(0,0,1,0|1,1) + p(0,0,1,1|1,1) + p(0,1,0,0|1,1) + p(0,1,0,1|1,1) + p(0,1,1,0|1,1)$$
$$+p(0,1,1,1|1,1) + p(1,0,0,0|0,1) - p(1,0,0,1|0,0) + p(1,0,0,1|0,1) + p(1,0,0,1|1,0) + p(1,0,1,0|1,1)$$
$$+p(1,0,1,1|1,1) + p(1,1,0,0|1,1) + p(1,1,0,1|1,1) + p(1,1,1,0|1,1) + p(1,1,1,1|1,1) \leq 0$$
$$\mathcal{I}_{61} = -1 + p(0,0,0,1|1,1) + p(0,0,1,0|1,1) + p(0,0,1,1|1,1) + p(0,1,0,0|1,1) + p(0,1,0,1|1,1) + p(0,1,1,0|1,1)$$
$$+p(0,1,1,1|1,1) + p(1,0,0,0|1,1) + p(1,0,0,1|0,0) - p(1,0,0,1|0,1) - p(1,0,0,1|1,0) + p(1,0,0,1|1,1)$$
$$+p(1,0,1,0|1,1) + p(1,0,1,1|1,1) + p(1,1,0,0|1,1) + p(1,1,0,1|1,1) + p(1,1,1,0|1,1) + p(1,1,1,1|1,1) \leq 0$$

SUPPLEMENTARY NOTES 4

Bilocal causality violation compared with entanglement swapping and CHSH

Our aim here is to analyze and compare two alternatives in order to witness the non-local character of the correlations in an entanglement swapping scenario: i) testing the CHSH inequality violations for the swapped quantum state between Alice and Charlie, ii) testing the bilocality inequality.

Entanglement swapping and CHSH protocol

Suppose that the two sources generates quantum states in the form described by equation (17). Our Bell-state measurement is expected to be able to discriminate $|\psi^-\rangle$ and $|\psi^+\rangle$ for one round (or $|\phi^-\rangle$ and $|\phi^+\rangle$ for a following round).
Post-selecting on singlet state outcomes, photons in stations A and C will be entangled in the following form:

$$\varrho_{AC\psi^-} = v\,|\psi^-\rangle\langle\psi^-| + (1-v)\cdot[\lambda\frac{|\psi^-\rangle\langle\psi^-|+|\psi^+\rangle\langle\psi^+|}{2} + (1-\lambda)\frac{\mathbb{I}}{4}] + \varrho_{ent.swap.-noise}(v,\lambda), \tag{38}$$

where

$$\varrho_{ent.swap.-noise}(v,\lambda) = \frac{1}{4}(\sqrt{v}-1)^2(1-\lambda)\lambda\cdot(|\phi^+\rangle\langle\phi^+|+|\phi^-\rangle\langle\phi^-|-|\psi^+\rangle\langle\psi^+|-|\psi^-\rangle\langle\psi^-|). \tag{39}$$

It is necessary to take into account how an imperfect Bell-state measurement (i.e. partial distinguishability in the BS) will affect the AC-state preparation. When the state in B is projected in $|\psi^+\rangle$, photons in A and C will end up in the state

$$\varrho_{AC\psi^+} = v\,|\psi^+\rangle\langle\psi^+| + (1-v)\cdot[\lambda\frac{|\psi^-\rangle\langle\psi^-|+|\psi^+\rangle\langle\psi^+|}{2} + (1-\lambda)\frac{\mathbb{I}}{4}] + \varrho_{ent.swap.-noise}(v,\lambda). \tag{40}$$

Imperfect Bell-state measurement will return, with probability $(1-p)/2$, the result associated with $|\psi^-\rangle$ (a_4) even though the state is projected in $|\psi^+\rangle$. When selecting singlet state in B via an imperfect Bell-state measurement, the final AC-state will thus be a convex sum of these two events, leading to

$$\varrho_{AC} = \frac{1+p}{2}\,\varrho_{AC\psi^-} + \frac{1-p}{2}\,\varrho_{AC\psi^+}$$

$$= v\,(\frac{1+p}{2}\,|\psi^-\rangle\langle\psi^-| + \frac{1-p}{2}\,|\psi^+\rangle\langle\psi^+|) + (1-v)[\lambda\frac{|\psi^-\rangle\langle\psi^-|+|\psi^+\rangle\langle\psi^+|}{2} + (1-\lambda)\frac{\mathbb{I}}{4}] + \varrho_{ent.swap.-noise}(v,\lambda). \tag{41}$$

Selecting $|\psi^+\rangle$ in station B and then applying $\sigma_z \otimes \mathbb{I}$ on the state shared by A and C will not modify this result since the imperfect Bell-state measurement will act in the same way, mixing only $|\psi^-\rangle$ and $|\psi^+\rangle$ and leading to equation (41).
The CHSH inequality can be written as

$$\mathcal{S} \equiv \frac{1}{2}|\,\langle A_0C_0 + A_0C_1 + A_1C_0 - A_1C_1\rangle\,| \leq 1. \tag{42}$$

If we choose the measurements given by

$$\begin{aligned} A_0 &= cos(\theta_0)\sigma_z + sin(\theta_0)\sigma_x, \\ A_1 &= cos(\theta_1)\sigma_z + sin(\theta_1)\sigma_x, \\ C_0 &= cos(\phi_0)\sigma_z + sin(\phi_0)\sigma_x, \\ C_1 &= cos(\phi_1)\sigma_z + sin(\phi_1)\sigma_x, \end{aligned} \tag{43}$$

we obtain

$$
\begin{aligned}
\mathcal{S}(\theta_0,\theta_1,\phi_0,\phi_1) = \frac{1}{2}| -\ & \beta(v,\lambda)\ \{cos(\theta_1)\cdot[cos(\phi_0)-cos(\phi_1)] + cos(\theta_0)\ [cos(\phi_0)+cos(\phi_1)]\} \\
-\ & \alpha(v,p)\ \{sin(\theta_1)[sin(\phi_0)-sin(\phi_1)] + sin(\theta_0)[sin(\phi_0)+sin(\phi_1)]\}|,
\end{aligned}
\tag{44}
$$

where $\alpha(v,p)$ and $\beta(v,\lambda)$ are the parameters introduced in (20). Our aim is to compare the bilocality violation with the best possible CHSH violation in an entanglement swapping scenario. We are thus looking for that choice of θ_0, θ_1 ϕ_0, ϕ_1 which maximizes $\mathcal{S}$, similarly to what was done in [2]. This can be achieved by setting angles as

$$
\begin{aligned}
\theta_0 &= 0, \quad \theta_1 = \tfrac{\pi}{2}, \\
\phi_0 &= \phi, \quad \phi_1 = -\phi,
\end{aligned}
\tag{45}
$$

which leads to

$$
\mathcal{S}(\theta_0=0,\theta_1=\tfrac{\pi}{2},\phi_0=\phi,\phi_1=-\phi) = |\ \beta(v,\lambda)\ cos(\phi) + \alpha(p,v)\ sin(\phi)|, \tag{46}
$$

In this case, CHSH maximization gives

$$
\begin{aligned}
\phi(\alpha(v,p),\beta(v,\lambda)) &= ArcTan(\frac{\alpha(v,p)}{\beta(v,\lambda)}), \\
\mathcal{S}_{max}(\alpha(v,p),\beta(v,\lambda)) &= \sqrt{\alpha(v,p)^2+\beta(v,\lambda)^2}.
\end{aligned}
\tag{47}
$$

It can be shown that equation (47) provides a full optimization of CHSH violation given the swapped state described in (41). In fact, a criterion by Horodecki *et al.* [4] asserts that the maximum $\mathcal{S}$ value allowed by QM given a quantum state ϱ can be computed as:

$$
\mathcal{S}_{max} = \sqrt{m_1^2+m_2^2}, \tag{48}
$$

where m_1^2 and m_2^2 are the two greatest eigenvalues of the matrix $T_\varrho^{\mathbf{T}} T_\varrho$, defined by

$$
T_\varrho \ :\quad t_{ij} \equiv Tr[\varrho(\sigma_i\otimes\sigma_j)], \tag{49}
$$

and where we took into account the definition in equation (42) which leads to a CHSH's bound equal to 1.
If ϱ_{AC} is written in the form given by equation (41), the computation of T_ϱ gives us that

$$
\begin{aligned}
& T_\varrho^{\mathbf{T}} T_\varrho \ \ eigenvalues: \\
& \{\ \alpha(v,p)^2,\ \alpha(v,p)^2,\ \beta(v,\lambda)^2\ \}.
\end{aligned}
\tag{50}
$$

It is easy to show that (47) and (48) are equivalent. In fact

$$
\begin{aligned}
& \beta(v,\lambda) = (\sqrt{v}(\lambda-1)-\lambda)^2 = v + 2(1-\sqrt{v})\sqrt{v}\lambda + (\sqrt{v}-1)^2\lambda^2 \geq v = \alpha(v,1) \geq \alpha(v,p), \\
& \forall\ 0\leq v\leq 1,\ 0\leq\lambda\leq 1,\ 0\leq p\leq 1,
\end{aligned}
\tag{51}
$$

and thus m_1^2 and m_2^2 will be given by $\beta(v,\lambda)^2$ and $\alpha(v,p)^2$.

Comparison between the protocols

The comparison between the bilocality and CHSH (for the swapped state) inequality violations is based on equations (26) and (47) which provide the maximum violation that can be obtained given a certain quantum state, for the two different protocols. This analysis is summarized in Fig. 3. It can be seen that if white noise is present (even partially), there is a wide region of v and p where only bilocality inequalities can be violated. In the case in which both inequalities can be violated, bilocality assumption provides a greater violation. In fact, since $\alpha(v,p)\leq 1$ and $\beta(v,\lambda)\leq 1$ are satisfied, equations (26) and (47) show that $0\leq\mathcal{S}_{max}\leq\mathcal{B}_{max}$ for all values of v, p and λ. Clearly, this could be useful in the future context of entanglement certification in complex quantum networks.

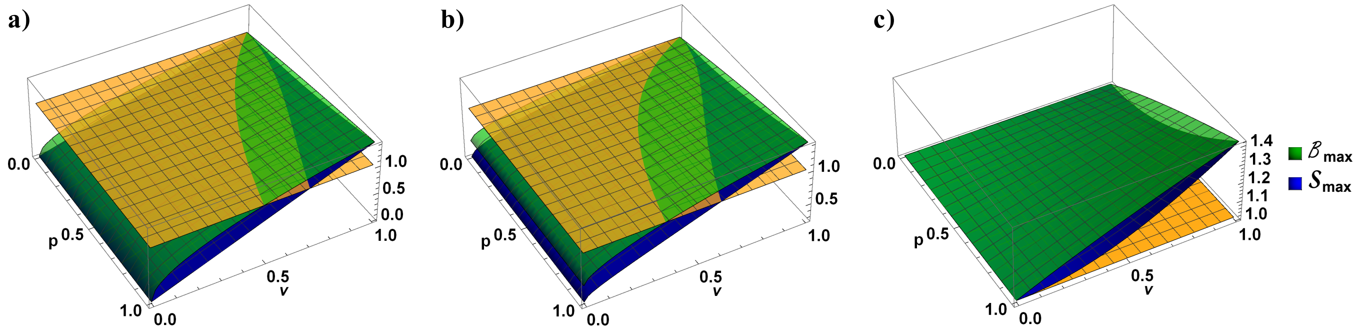

Supplementary Figure 3: Comparison between CHSH protocol and bilocality violation. Maximum possible violation varying v and p given an entanglement swapping and CHSH protocol (blue surface) or bilocality assumption (green surface). Orange plane shows violation threshold. Figures represent the cases when: **(a)** $\lambda = 0$ (i.e. white noise), **(b)** $\lambda = 1/2$ (i.e. half white half colored noise) and **(c)** $\lambda = 1$ (i.e. colored noise).

Excursus on the two parties Bell scenario

It is interesting to investigate how CHSH maximization works when dealing with the Bell scenario depicted in Fig. 1-a of the main text. This is not directly related to our main argument, which concerns a quantum network of three parties, but could be very useful in order to characterize the best measurements settings in case of a simpler Bell scenario (for different noises involved). Suppose that A and C directly shares a state analogous to the one defined in equation (17), with parameters v and λ.

Performing the measurements defined in equation (43) leads to:

$$\mathcal{S}_{direct} = \frac{1}{2}|\ \gamma(v,\lambda)[cos(\theta_0)(cos(\phi_0)+cos(\phi_1)) + cos(\theta_1)(cos(\phi_0)-cos(\phi_1))] + v[sin(\theta_0)(sin(\phi_0)+sin(\phi_1)) \\ +sin(\theta_1)(sin(\phi_0)-sin(\phi_1))]\ |, \tag{52}$$

where we defined

$$\gamma(v,\lambda) = \lambda - v(\lambda - 1). \tag{53}$$

The application of the settings described in equation (45) gives that

$$\mathcal{S}_{direct} = |\gamma(v,\lambda)\ cos(\phi) + v\ sin(\phi)|, \tag{54}$$

whose maximization is given by

$$\phi(v,\gamma(v,\lambda)) = ArcTan(\frac{v}{\gamma(v,\lambda)}), \\ \mathcal{S}_{direct\,-\,max}(v,\delta(v,\lambda)) = \sqrt{v^2+\gamma(v,\lambda)^2}. \tag{55}$$

The application of the Horodecki criterion (equation 48) to this case shows that equation (55) provides a complete and full optimization of CHSH violation, given a quantum state with both colored and white noise. For the case $\lambda = 1$ this equation is reduced to $\sqrt{1+v^2}$, which agrees with the results pointed out in [5] for a system of interacting spins.

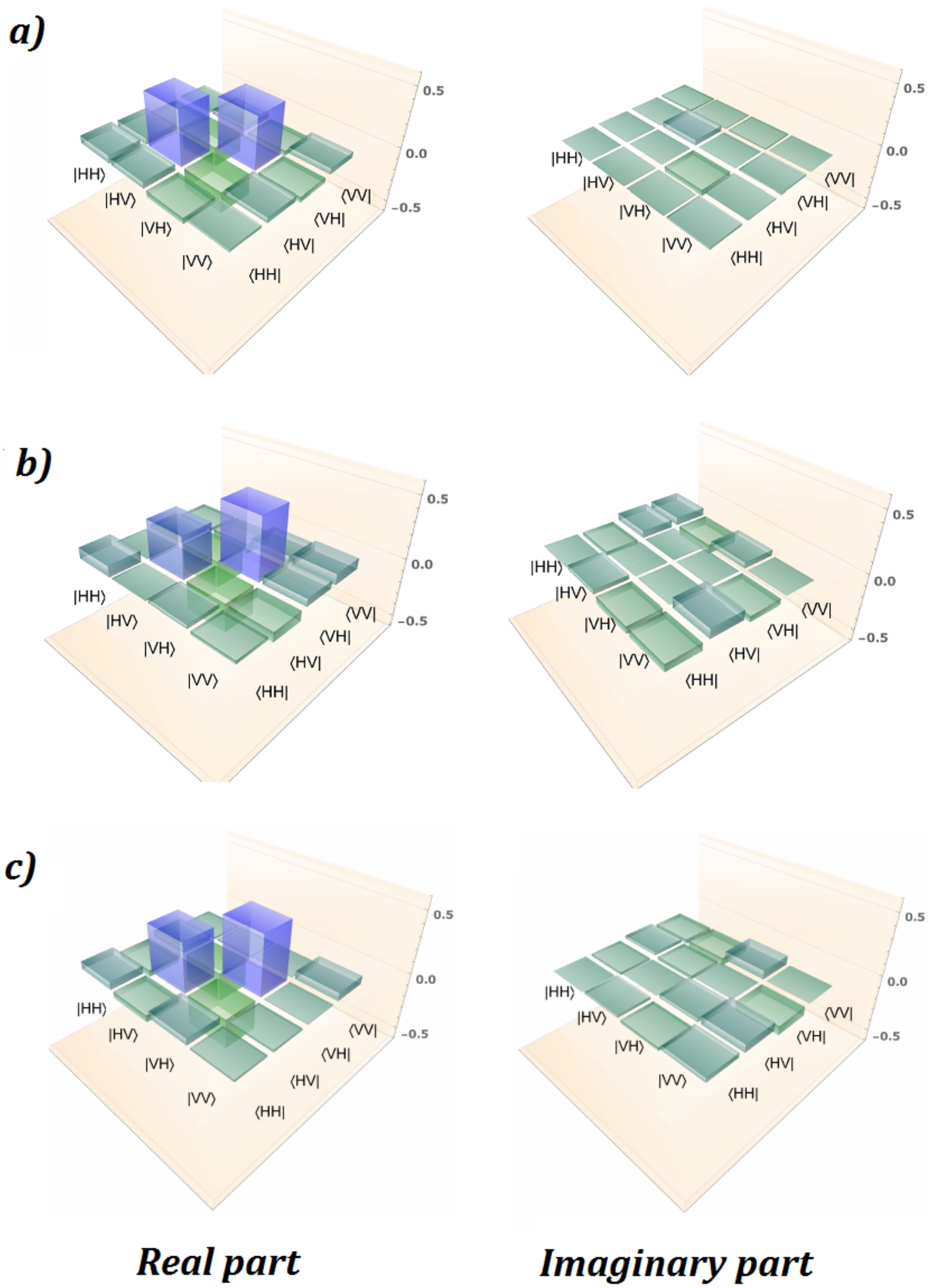

Supplementary Figure 4: Full quantum state tomographies for the blue points in Fig. 4-b of the main text belonging to the light green region. Tomography **(a)**, **(b)** and **(c)** corresponds respectively to the point with the lowest, intermediate and higher value of CHSH violation.

Experimental estimation of maximum CHSH violation

The maximum CHSH violation allowed for the state shared between Alice and Charlie after entanglement swapping was computed (Fig. 4-b of the main text) exploiting the Horodecki criterion (Ref. [4]) defined in formula (48). We performed several quantum state tomographies for Alice and Charlie's photons, conditioned on Bob's outcome $|\psi^-\rangle$, and then applied this criterion. The blue points in Fig. 4-b of the main text were obtained exploiting a full quantum state tomography, which needs all the 9 combinations of σ_x, σ_y, and σ_z for the two photons. In Fig. 4 we show the results for the three points of the dataset which do not violate CHSH inequality between Alice and Charlie but are not compatible with a bilocal model.. The measured fidelities and concurrences for the three tomographies are presented in Supplementary Table 1. The exhibit values are consistent with the quantum state modeling shown in equation 41, for the estimated p for this point.

State	Fidelity	Concurrence
(a)	0.906 ± 0.024	0.350 ± 0.014
(b)	0.941 ± 0.020	0.391 ± 0.015
(c)	0.965 ± 0.019	0.262 ± 0.018

Supplementary Table 1: Fidelity and Concurrence values for the blue points in Fig. 4-b of the main text belonging to the light green region. State **(a)**, **(b)** and **(c)** corresponds respectively to the point with the lowest, intermediate and higher value of CHSH violation.

When dealing with fourfold coincidence events (i.e. after entanglement swapping), a full quantum state tomography requires several hours of continuous measurement. To reduce the experimental effort, it is possible to perform a different tomography which relies on the state modeling, that is, assuming that the state has the form:

$$\varrho = \begin{pmatrix} a & 0 & 0 & 0 \\ 0 & b & e & 0 \\ 0 & e & c & 0 \\ 0 & 0 & 0 & d \end{pmatrix}, \tag{56}$$

where a, b, c, d and e are free real parameters. Experimental estimations of these parameters can be obtained by performing only the two measurements $\sigma_z^A \otimes \sigma_z^C$ and $\sigma_x^A \otimes \sigma_x^C$. Comparing the states given by the partial quantum tomography and the full quantum tomography for these three points we obtained fidelities higher than 0.9, which show that the results for these two protocols are in good agreement. Data corresponding to partial tomographies are shown as red points in Fig. 4-b of the main text.

SUPPLEMENTARY REFERENCES

[1] Mattle, K., Weinfurter, H., Kwiat, P.G. & Zeilinger, A. Dense Coding in Experimental Quantum Communication. *Phys. Rev. Lett.* **76**, 4656-4659 (1996) .

[2] Cabello, A., Feito, A. & Lamas-Linares, A. Bell's inequalities with realistic noise for polarization-entangled photons. *Phys. Rev. A* **72**, 052112 (2005) .

[3] Branciar, C., Rosset, D., Gisin, N. & Pironio, S. Bilocal versus nonbilocal correlations in entanglement-swapping experiments. *Phys. Rev. A* **85**, 032119 (2012) .

[4] Horodecki, R., Horodecki, P., & Horodecki, M. Violating Bell inequality by mixed spin-12 states: necessary and sufficient condition. *Physics Letters A* **200**, 340-344 (1995) .

[5] Levkovich-Maslyuk, R., Two destructive effects of decoherence on Bell inequality violation. *Phys. Rev. A* **79**, 054101 (2009) .

[6] Pitowsky, I., Correlation polytopes: Their geometry and complexity. *Mathematical Programming* **50**, 395 (1991) .

[7] Christof, T., & Lobel, A. PoRTA-PoLYHEDRON Representation Transformation Algorithm (2009)
